# Supplementary material for: Interaction of TSG101 with the PTAP Motif in Distinct Locations of Gag Determines the Incorporation of HTLV-1 Env into the Retroviral Virion
Source: Int J Mol Sci. 2023 Nov 20;24(22):16520. doi: 10.3390/ijms242216520 (PMC10671467; doi:10.3390/ijms242216520)
Supplement: Supplementary file 1 [file ijms-24-16520-s001.zip › ijms-2665053-supplementary.pdf]

Supplementary Figures

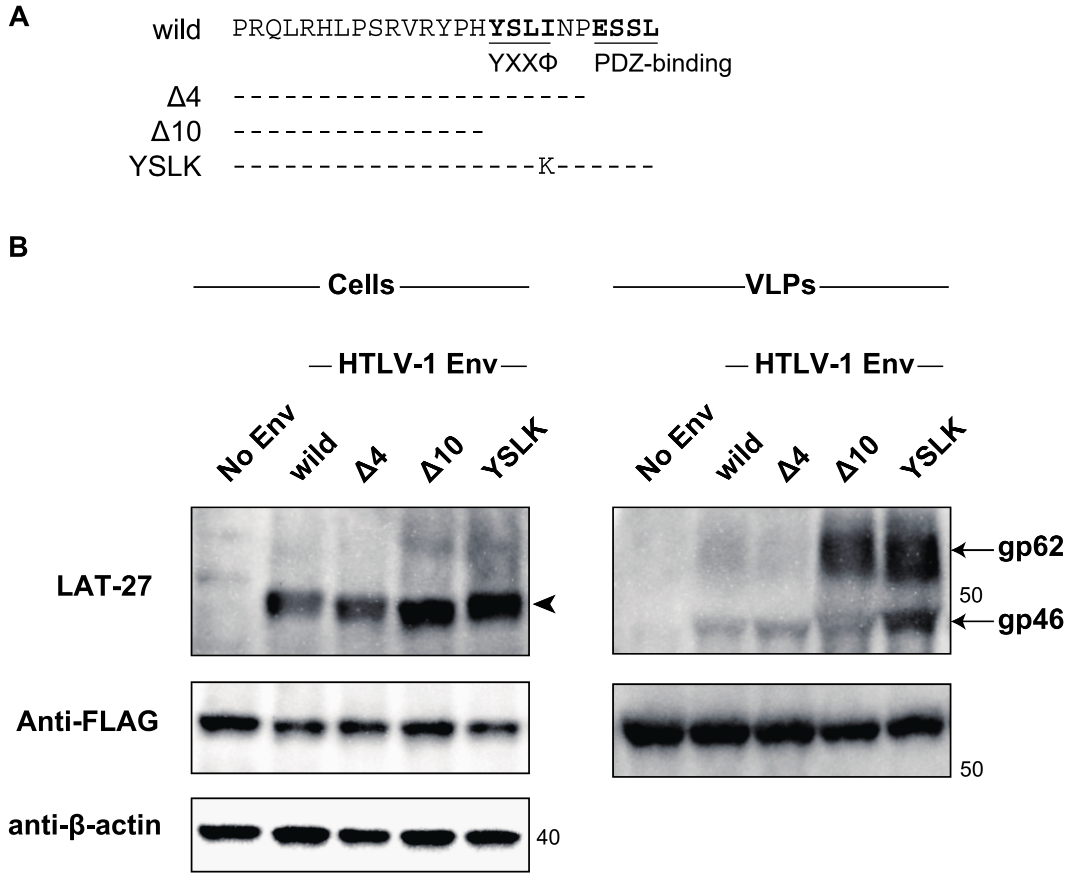

Supplementary Figure S1. Effect of HTLV-1 Env cytoplasmic domain mutants on incorporation into HIV-1-Gag VLPs. (A) Schematic representation of deletions and mutation in the cytoplasmic tail of HTLV-1 Env. The amino acid sequences of wild-type cytoplasmic domain of HTLV-1 Env are indicated as Δ4, Δ10, and YSLK. A dash denotes amino acid identity. (B) The cell lysates and VLPs produced from 293T cells transfected with HTLV-1 Env deletion and mutants with HIV-1-Gag-FLAG were analyzed by western blotting using LAT-27, anti-FLAG, and anti-β-actin. The positions of the molecular mass marker (kDa) are indicated on the right. The arrowhead shows the ~53-kDa size of the Env protein.

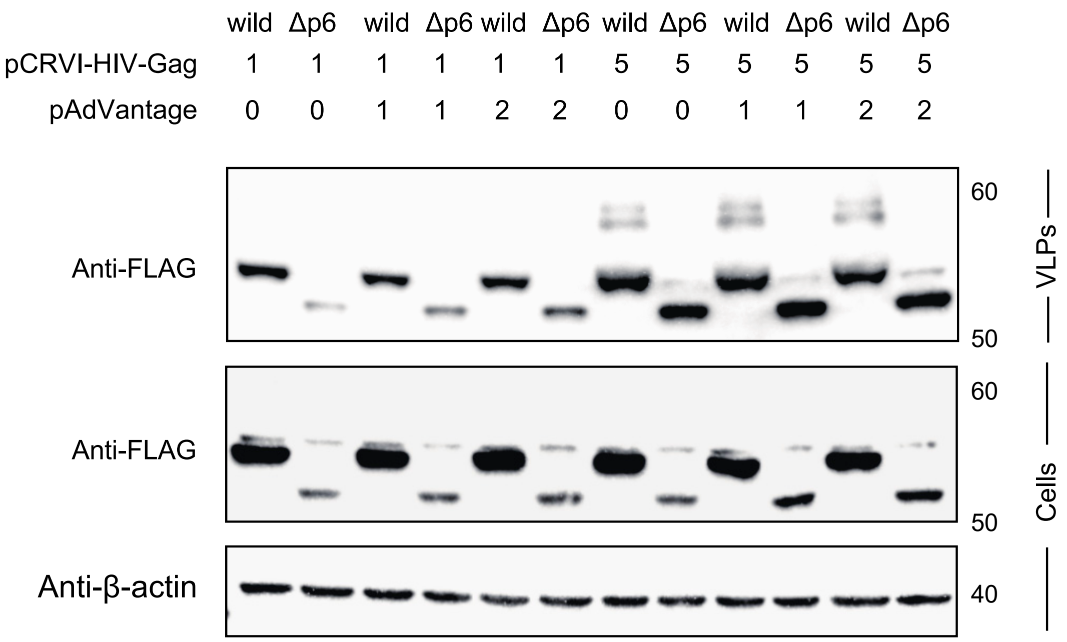

Supplementary Figure S2. Effect of overexpression of HIV-Gag and pAdVantage on VLP production. (A) Cell lysates and VLPs produced from 293T cells transfected with different amounts of HIV-1-Gag wild-type or p6 deletion mutants with different amounts of pAdVantage were analyzed by western blotting using anti-FLAG and anti-β-actin antibodies. The positions of the molecular mass marker (kDa) are indicated on the right.

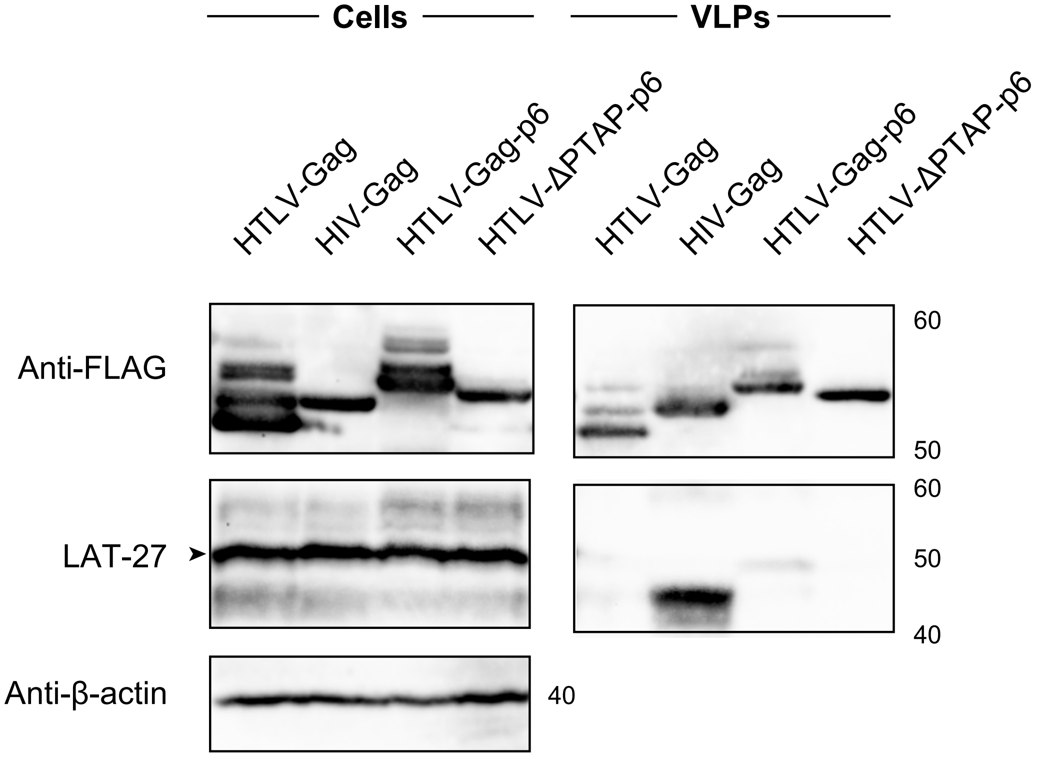

Supplemental Figure S3. Efficiency of HTLV-1 Env incorporation into VLPs produced by HTLV-1 Gag fused with HIV-1 p6. The cell lysates and VLPs produced from 293T cells transfected with HTLV-1 Env and HTLV-1-Gag or -ΔPTAP-Gag fused with HIV-1 p6 were analyzed by western blotting using anti-FLAG, LAT-27, and anti-β-actin antibodies. The positions of the molecular mass marker (kDa) are indicated on the right.

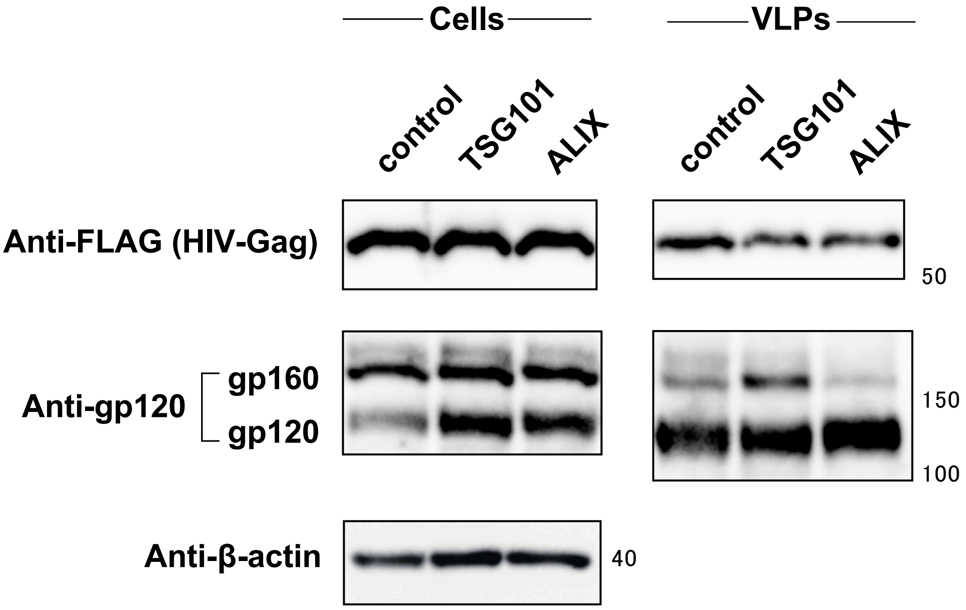

Supplementary Figure S4. Incorporation of HIV-1 Env in HIV-1-Gag VLPs produced in 293T cells with TSG101 or ALIX knockdown. TSG101 or ALIX knockdown 293T cells were transfected with HIV-Gag-FLAG and HIV-1 Env. The cell lysate and VLPs were recovered after 48 h of transfection and analyzed by western blotting using anti-FLAG, anti-gp120, and anti-β-actin antibodies. The 293T cells transduced with a control shRNA vector (control) were used as the control. The positions of the molecular mass marker (kDa) are indicated on the right.
